# Key Signals Produced by Gut Microbiota Associated with Metabolic Syndrome, Cancer, Cardiovascular Diseases, and Brain Functions

**DOI:** 10.3390/ijms262110539

**Published:** 2025-10-29

**Authors:** Leon M. T. Dicks

**Affiliations:** Department of Microbiology, Stellenbosch University, Stellenbosch 7600, South Africa; lmtd@sun.ac.za

**Keywords:** gut microbiota, molecular signals, metabolic syndrome, cancer, cardiovascular diseases, brain functions

## Abstract

Gut microbiota have a significant impact neurotransmitters, short-chain fatty acids (SCFAs), immune signaling molecules, and gut hormones. These signaling molecules interact with receptors on the gut wall, immune cells, or the enteric nervous system (ENS), and reach the central nervous system (CNS) via the Vagus nerve (VN). SCFAs interact with G protein-coupled receptors (GPCRs), Toll-like receptors (TLRs), and proliferator-activated receptors (PPARs), influencing inflammatory reactions, gut motility, nutrient absorption, hormone secretion, neurochemical signaling, and brain functions. Olfactory receptor OR51E1 influences blood pressure, vascular reactivity, and arterial stiffness. Activation of the brainstem nucleus tractus solitarius (NTS) by glucagon-like peptide 1 (GLP-1) influences mood, cognition, and gastrointestinal motility. Prolactin-releasing peptide (PrRP) binds to its receptor (PrRPR), suppressing food intake, and regulating stress, cardiovascular reactions, and circadian rhythms. In-depth studies on how gut microbiota control cognitive behavior, mood, and neuropsychiatric disorders are lacking. G protein receptor 119 (GPR119) suppresses appetite and may find an application in the treatment of type 2 diabetes and obesity. The binding of butyrate to nuclear factor kappa B (NF-κB) and proliferator-activated receptor γ (PPARγ) regulates the production of pro-and anti-inflammatory cytokines. This suppresses protein CD36, preventing the uptake of oxidized low-density lipoprotein (ox-LDL) and cardiovascular diseases (CVDs). This review focuses on a few prominent health conditions related to CVDs, i.e., metabolic syndrome (MetS), cancer, and brain functions. Information in this review is based on animal and preclinical studies published in repositories such as PubMed, the National Institutes of Health (NIH), NIH PubChem, ScienceDirect, MDPI, Frontiers, Cell Press, and the CAS Content Collection.

## 1. Introduction

**Figure 1:**
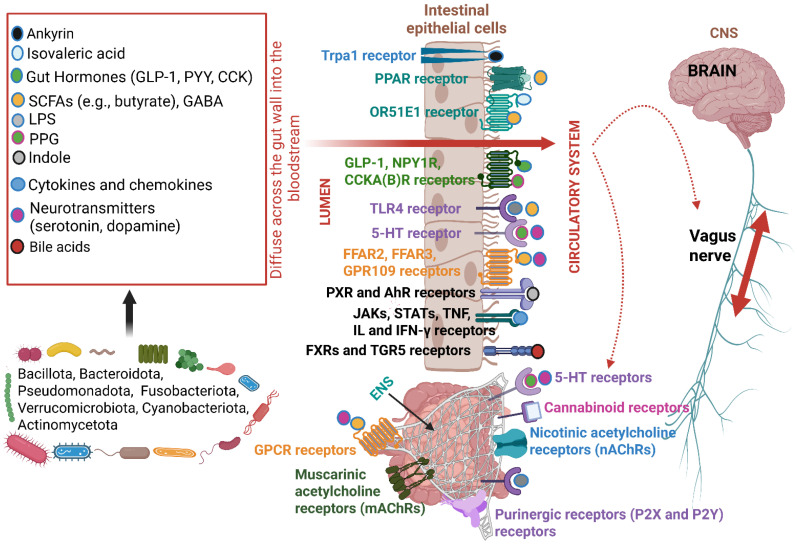
Metabolites, intermediate compounds, and signaling molecules (in red box), produced by gut microbiota, cross the gut–blood barrier (intestinal epithelium) and bind to receptors on or embedded into intestinal epithelial cells (IECs) and neurons of the enteric nervous system (ENS). Most of these molecules are produced by bacteria in the large intestinal tract. Signals generated from the receptor–ligand interactions reach the central nervous system (CNS) via the Vagus nerve (VN). Messages interchanged between the brain, gut wall, and ENS are referred to as the gut–brain axis (GBA). The horizontal red arrow denotes diffusion or active transport of molecules across the gut wall. The double arrow indicates bi-directional communication (GBA). The dotted red arrows denote signals sent to neurons of the ENS (depicted as the blue net-like structure) and the VN. GLP-1 = glucagon-like peptide 1; PYY = peptide tyrosine-tyrosine; CCK = cholecystokinin; SCFAs = short-chain fatty acids; GABA = gamma-aminobutyric acid; LPS = lipopolysaccharide; PPG = pseudopeptidoglycan; Trpa1 = transient receptor potential ankyrin 1; PPAR = proliferator-activated receptor; OR51E1 = an olfactory receptor 51E1; NPY1R = neuropeptide Y receptor type 1; CCKA(B)R = cholecystokinin A or B receptor; TLR4 = Toll-like receptor 4; 5-HT = 5-hydroxytryptamine (serotonin); FFAR = free fatty acid receptor; GPR = G protein receptor (also known as GPCR, G protein-coupled receptor); PXR = pregnane X receptor; AhR = aryl hydrocarbon receptor; JAKs = Janus kinases; STATs = signal transducers and activators of transcription; TNF = tumor necrosis factor; IL = isoleucine; IFN-γ = interferon gamma; FXRs = farnesoid X receptors; TGR5 = Takeda G protein-coupled receptor 5. This presentation was created in Biorender. LMT Dicks. (2025) https://app.biorender.com/illustrations/68d153c9d08acd97771ba1f (accessed on 23 September 2025).

The adult human gut microbiome consists of 3.8 × 10^13^ (200 g) bacteria, composed of members from the phyla Bacillota (previously Firmicutes), Bacteroidota (previously Bacteroidetes), Pseudomonadota (previously Proteobacteria), Fusobacteriota (previously Fusobacteria), Verrucomicrobiota (previously Verrucomicrobia), Cyanobacteriota (previously Cyanobacteria), and Actinomycetota (previously Actinobacteria) (Figure 1) [1]. Bacillota and Bacteroidota are predominant [2]. The gut microbiome is also composed of fungi, of which *Candida*, *Saccharomyces*, *Malassezia*, and *Cladosporium* are the most represented [2], and viruses such as *Enterovirus* from the family *Picornaviridae* [3], *Rotavirus* [4] and *Norovirus* [5] from the families *Reoviridae* and *Caliciviridae*, respectively, as well as *Picobirnaviridae*, *Astroviridae*, *Adenoviridae*, *Anelloviridae*, *Cycloviridae*, and *Parvoviridae* [6]. Shifts in bacterial populations, especially Bacillota, Bacteroidota, Pseudomonadota, and Actinomycetota, depend on diet, age, medication, hormone levels, and stress [6,7,8,9], the availability of nutrients, peptide (and enzyme) production, and the state of the host’s immune system [10]. It is thus conceivable that most research on human health focuses on preventing dysbiosis and maintaining the immune system in a balanced state.

Molecules generated in response to changes in the physiological conditions of gut microbiota, intestinal epithelial cells (IECs), and endothelial cells have a broad impact on our health, and some of these may be utilized in the early detection of certain abnormalities. Intermediate compounds and end metabolites produced by gut microbiota either diffuse across the gut wall or are actively transported into the bloodstream, where they interact with receptors on IECs, endothelial cells, and immune cells. Signals generated from these interactions reach the enteric nervous system (ENS) and central nervous system (CNS) via the Vagus nerve (VN) [11,12] (dashed lines, Figure 1). Most of these signaling molecules (color-coded receptors in boxed square, Figure 1) are recognized by pattern recognition receptors (PRRs) and damage-associated molecular patterns (DAMPs) located on, or embedded into, the intestinal epithelium [1,10,13,14,15,16]. Examples of receptors attached to the ENS are shown at the bottom of Figure 1.

Ankyrin interacts with the transient receptor potential ankyrin 1 (Trpa 1), located on IECs (Figure 1), vascular endothelial cells, and respiratory cells. Ankyrin repeats interact at the N-terminal of Trpa 1 and are linked to reactive cysteine residues coupled to the first of six transmembrane domains [17]. The cysteine residues are chemically modified by reactive oxygen species (ROS) and other electrophiles produced during oxidative stress. This leads to the formation of disulfide bonds and covalent changes, which activate channel formation, resulting in the opening of the pore between transmembrane domains 5 and 6 through which oxidative stress byproducts can pass [17]. The activity of the Trpa 1 channel is modulated by negatively charged ligands, including phosphoinositides and inorganic polyphosphates [18], which interact with a yet unidentified positively charged domain in the C-terminal region. The C-terminal of Trpa 1 contains a calcium-binding region and is connected to the cytoskeleton. The C-terminal is sensitive to voltage and chemical changes and is involved in protein–protein interactions that play a vital role in directing the channel to the plasma membrane. The C-terminal may thus interact with negatively charged molecules such as lipids to modulate the protein’s activity, e.g., in the assembling of hetero-tetramer complexes [18].

Trpa 1 anchored to the cytoskeleton releases neuropeptides such as substance P (SP) and CGRP (calcitonin gene-related peptide) when activated by inflammatory mediators [18]. Ankyrin also plays a role in tissue repair and intestinal motility [19]. By regulating the binding of ankyrin to Trpa 1, allergies, asthma, chronic pain, and gastrointestinal disorders may be treated [20]. Indole derivatives produced by *Edwardsiella tarda* activate Trpa1, leading to the release of serotonin (5-HT), which modulates gut motility [21]. The ankyrin-containing protein Cj1386 produced by *Campylobacter jejuni* is essential to colonize IECs and is involved in transporting heme to the enzyme catalase, which is crucial for protecting the bacterium from oxidative stress [22].

Isovaleric acid, produced during the breakdown of leucine, leads to the production of acetyl-CoA, an intermediate in the synthesis of cholesterol and fatty acids [23]. Isovaleric acid and short-chain fatty acids (SCFAs) interact with the orthosteric-binding site of olfactory receptor OR51E1 (equivalent to olfactory receptor 558, Olfr588, in mice), located on IECs (Figure 1). This triggers a cascade of downstream signaling reactions that involves the activation of the G protein Gα_olf_, leading to the stimulation of adenylyl cyclase (ACIII), an increase in intracellular cyclic AMP (cAMP) levels, and stimulation of protein kinase A (PKA) and other downstream effectors [23]. The stimulation of ACIII catalyzes the conversion of ATP (adenosine triphosphate) to cAMP. The increase in cAMP inhibits the influx of Ca^2+^ in vascular smooth muscle cells (VSMCs), leading to vasodilation (lower blood pressure) [23] and the release of renin [24]. *Bacteroides* and *Clostridium* species are major producers of isovaleric acid [25].

Isovaleric acid and other branched-chain fatty acids (BCFAs) produced by gut microbiota increase protein SUMOylation in intestinal cells in a pH-dependent manner. These BCFAs inactivate intestinal deSUMOylases, promote the hyperSUMOylation of nuclear matrix-associated proteins, inhibit the NF-κB pathway, decrease pro-inflammatory cytokine expression, and promote intestinal epithelial integrity [25]. SCFAs, predominantly produced by *Bacteroides*, *Prevotella*, and *Actinobacteria*, bind to free fatty acid receptors (FFARs), also referred to as G protein-coupled receptors (GPCRs or G protein receptors, GPRs), Toll-like receptors (TLRs), and proliferator-activated receptors (PPARs) on IECs, endothelial cells and neurons of the ENS (Figure 1), influencing inflammatory reactions, regulating signaling pathways, and controlling energy homeostasis [14]. In healthy individuals, the plasma levels of SCFAs range from 50 to 150 µM, with acetate, propionate, and butyrate in the colon at a ratio of 60:20:20 [24]. The half-maximal effective concentration (EC_50_) for GPR41 and GPR43 varies, with acetate, propionate, and butyrate detected in the micromolar range. The EC_50_ for acetate, propionate, and butyrate interacting with CPR41 range from 393 to 1072 µM, 6 to 127 µM, and 33 to 158 µM, respectively [24]. EC_50_ values for acetate, propionate, and butyrate adhered to for GPR43 range from 35 to 431 µM, 14 to 290 µM, and 28 to 371 µM, respectively, but are highly dependent on diet [24]. The ratio of acetate/propionate/butyrate varies from 40:26:34 to 78:17:5 [24]. The circulating (plasma) levels of acetate range from 50 to 500 µmol/L [24]. It is thus likely that physiological changes in acetate may alter GPR41 or GPR43 signaling.

The interaction of lipopolysaccharides (LPSs) with Toll-like receptor 4 (TLR4) involves a series of steps [26]. LPS is first bound by coreceptors CD14 and MD2 before it binds to TLR4. This causes the dimerization of TLR4 and activation of the MyD88 (myeloid differentiation primary response 88)-dependent and the TRIF [Toll/interleukin-1 receptor (TIR)-domain-containing adapter-inducing interferon-β]-dependent pathways. These pathways activate transcription factors such as Nuclear Factor-kappa B (NF-κB) and Interferon Regulatory Factor (IFR), which drive the production of pro-inflammatory cytokines [27]. Pro-inflammatory LPSs, mostly produced by Gram-negative bacteria (e.g., *Escherichia coli*), trigger inflammatory reactions and damage the gut wall. LPS that leaks into the bloodstream contributes to conditions such as type 2 diabetes, chronic kidney disease, and inflammatory bowel disease (IBD) [28].

Indole interacts with aryl hydrocarbon receptors (AHRs) and pregnane X receptors (PXRs) on the gut wall (Figure 1), regulating gut motility, nutrient absorption, hormone secretion, neurochemical signaling, and brain functions. Activated AHR translocates to the nucleus, binds with the ARNT (AHR/AHR nuclear translocator) protein, and then to target genes, including those involved in detoxification and immune responses [29].

Neurotransmitters (serotonin and dopamine) bind to FFARs on IECs and neurons of the ENS (Figure 1), to regulate inflammation, feeding behavior, gut integrity, and hormone synthesis [30,31,32,33]. Serotonin binds to 5-HT receptors on IECs and neurons of the ENS (Figure 1). Serotonin can modulate dopamine neuron activity and the release of dopamine by activating serotonin receptors such as 5-HT1A, 5-HT1B, 5-HT2A, and 5-HT3). Serotonin receptor 5-HT2C inhibits dopamine release [34]. *Lactobacillus, Bifidobacterium*, *Clostridium*, and *Ruminococcus* are typical serotonin producers. *Clostridium sporogenes* produces serotonin indirectly, by producing tryptamine, which stimulates enterochromaffin cells to produce their own serotonin [30].

Gut hormones such as glucagon-like peptide 1 (GLP-1), peptide tyrosine-tyrosine (PYY), and cholecystokinin (CCK), bind to GLP-1, NPY1, and CCKA(B) receptors, respectively, on IECs (Figure 1) [35]. These hormones are secreted in response to nutrients and regulate glucose metabolism and insulin sensitivity [36]. Fatty acid receptors GPR119 and GPR55, activated by fatty acid ethanolamides, such as oleoylethanolamide (OEA) and almitoylethanolamide (PEA), and phospholipids, such as lysophosphatidylcholine (LPC), regulate glucose homeostasis and increase the secretion of insulin and GLP-1 [24,30]. The fatty acids oleoylethanolamide and lysophosphatidylcholine are agonists of GPR119. However, supplementation with butyrate showed potential for treating type 2 diabetes and metabolic dysfunction-associated steatotic liver disease (MASLD) [37]. Butyrate increased the release of insulin from pancreatic β-cells in a glucose-dependent manner and increased the release of GLP-1 and glucose-dependent insulinotropic polypeptide (GIP) from L- and K-cells [38]. By increasing GLP-1, GPR119 agonists can lead to reduced food intake, and a decrease in body weight. Although treatment of type 2 diabetes and MASLD with butyrate supplementation is promising, extended research must be conducted to evaluate the consistency of the data thus far obtained from clinical trials, determine the most effective delivery method, and evaluate treatment over an extended period to evaluate the glycemic data. Most of these studies have been performed on animals. The safety and long-term effect of GPR119 agonists in humans is uncertain.

The secretion of GLP-1 and PYY from intestinal cells is stimulated by the interaction of certain medium-chain fatty acids, such as decanoic acid, with OR51E1 [39]. The secretion of GLP-1 and PYY depends on the levels of decanoic acid, as shown by blocking the OR51E1 receptors in L-cell lines [39]. Findings from this research suggested that OR-mediated events in GLP-1 and PYY secretion may be applied in treating diabetes and CVDs. Jovancevic et al. [40] showed that activation of OR51E1 by MCFA induces negative inotropy in human explanted heart preparations. The authors also showed negative chronotropy in stem cell-derived cardiomyocytes, which could be reversed by the OR51E1 antagonist 2-ethylhexanoic acid. These results suggest that OR51E1 may play a role in the metabolic regulation of cardiac functions. However, as stated by the authors, further research on animal models and patients is required to have conclusive evidence. High concentrations of MCFAs may, however, negatively affect cardiac function and contribute to diabetic cardiomyopathy by causing the heart to rely excessively on fat for energy. This may lead to the accumulation of toxic lipid byproducts, oxidative stress, impaired glucose metabolism, mitochondrial dysfunction, and damage to VSMCs [41].

The binding of butyrate to PPAR (Figure 1), specifically PPARγ, suppresses the production of pro-inflammatory cytokines by stimulating the activity of IkBα, an inhibitor of the NF-κB pathway [14]. With a decrease in NF-κB, the levels of anti-inflammatory cytokines, such as IL-10, increase [1,14]. The production of IL-10 is further increased as butyrate binds to FFAR3, following the suppression of interferon gamma (IFN-γ) and TLR2 [14]. Binding of butyrate to FFAR3 also suppresses the NLRP3 inflammasome, resulting in a decrease in pro-inflammatory cytokines [14]. Butyrate anchored to FFAR2 on colonocytes regulates the integrity of IECs, endocrine and immune pathways, the autonomic nervous system (ANS), and CNS, as reviewed by Dicks [1]. The binding of butyrate to GPCR109A (hydroxycarboxylic acid receptor 2, HCAR2) (Figure 1) on colonic cells stimulates macrophages and dendritic cells, leading to the differentiation of regulatory T cells (Tregs) and the production of anti-inflammatory cytokines [33]. GPCR109A also serves as a docking station for neurotransmitters (Figure 1) and niacin (vitamin B) [14].

Neurons of the ENS have receptors for serotonin, cannabinoids (e.g., endocannabinoids produced by humans and phytocannabinoids from plants), nicotinic acetylcholine that mediate fast synaptic transmissions between neurons and at neuromuscular junctions, purinergic molecules (purine nucleotides and nucleosides, such as adenosine and ATP), muscarinic acetylcholine that regulates heart rate, smooth muscle contractions, and gland secretions, and SCFAs (bottom image, Figure 1).

SCFAs are arguably the most important signaling molecules produced by gut microbiota. Most SCFAs are produced in the colon by *Bifidobacterium*, *Lactobacillus*, *Lachnospiraceae*, *Blautia*, *Coprococcus*, *Roseburia*, *Faecalibacterium*, *Clostridium*, and *Eubacterium* [1]. Butyrate, the main SCFA, supplies 70% of the energy requirements of colonic epithelial cells and regulates the expression of tight-junction proteins, thus maintaining gut permeability. Most SCFAs are transported across the gut wall in dissociated form by an unknown HCO_3_^−^ exchanger, monocarboxylate transporter-1 (MCT1) or sodium-coupled monocarboxylate transporter-1 (SMCT1). SCFAs may, however, diffuse across IEC membranes and enter the bloodstream in a non-ionized form. Butyrate stimulates the differentiation of colonic regulatory T cells, the production of reactive oxygen species (ROS), and aryl hydrocarbon receptors (AhRs) [10,13,14,15]. AhR also serves as a docking station for indole (Figure 1) and downregulates intestinal inflammation, inflammatory bowel diseases (IBD), including Crohn’s disease and ulcerative colitis (UC), celiac disease, liver disease, and neurological diseases (reviewed by Dicks [1]). An increase in AhR leads to a decrease in interferon gamma (IFNγ), isoleucine (IL)-6, IL-12, IL-7, IL-17, and tumor necrosis factor (TNF), a decline in microbial translocation and fibrosis, an increase in regulatory mechanisms such as IL-10, IL-22, prostaglandin E2, and Foxp3 (scurfin), and the production of antimicrobial peptides [1]. Free fatty acids (FFAs) from the lipolysis of triacylglycerol (TAG) bind to albumin in the bloodstream [27]. Less glucose is metabolized when FFAs are used as energy [42]. Excess TAG stored in adipose tissue in muscle, and the liver leads to an increase in lipotoxic intermediates such as diacylglycerol (DAG) [43] and ceramide, ultimately leading to insulin resistance [44].

The binding of butyrate to TLR4 increases the production of nicotinamide adenine dinucleotide phosphate (NADPH) and activates the mitogen-activated protein kinase (MAPK) and NF-κB pathways [14]. The latter increases the expression of endothelial nitric oxide synthase (eNOS), which leads to an increase in the production of nitric oxide (NO), a potent vasodilator. NF-κB also increases the production of pro-inflammatory cytokines TNF-α, IL-6, and IL-1β [1,14].

Primary bile acids (cholic acid, CA, and chenodeoxycholic acid, CDCA), produced in the liver, are absorbed by IECs. However, a portion of primary bile acids is converted to secondary bile acids (deoxycholic acid, DCA, and lithocholic acid, LCA) by gut microbiota [1,14]. Bile acids act as signaling molecules that activate receptors such as farnesoid X receptors (FXRs) and Takeda G protein-coupled receptor 5 (TGR5), located in the liver, intestine (Figure 1), muscle, and adipose tissue [45]. Signals generated from these interactions influence glucose and lipid metabolism [46,47]. In a murine model, bile acids interacting with TGR5 increased GLP-1 and PYY secretion [48].

Enterochromaffin cells, a subtype of EECs, produce more than 90% of the serotonin from the metabolism of tryptophan, in addition to other neurotransmitters such as the catecholamines dopamine (DA), norepinephrine (NE, noradrenaline), and epinephrine (adrenalin) [49]. Tryptophan is also converted by gut microbiota to indole and indole derivatives (e.g., indole propionic acid, indole acetic acid, tryptamine, and serotonin) that binds to AhRs and pregnane X receptors (PXRs) (Figure 1), modulating immune homeostasis, gut barrier integrity, and neurobiological processes [50]. Trace amines, histamine, and tyramine interact with the ENS and CNS via the gut–brain axis (GBA) [51].

Other signaling molecules include toxins, microbial cell components (e.g., peptidoglycan and flagellin), autoinducers (AIs), homoserine lactones (HSLs), 2-heptyl-3-hydroxy-4(1H)-quinoline; 2-heptyl-4-hydroxyquinoline (HHQ), quorum-sensing peptides (QSPs), peptide pheromone Agr, and pore-forming toxins such as hemolysins, leucocidins, and phenol-soluble modulins (psms), reviewed by Dicks [13]. Bacterial toxins such as fragilysin (BFT), produced by *Bacteroides fragilis*, damage tight junction proteins and increase gut permeability [52]. Uremic toxins, produced from the metabolism of choline, betaine, tyrosine, and tryptophan, increase inflammation and damage the intestinal barrier [53]. Toxins such as diphtheria toxin (*Clostridium diphtheriae*), enterotoxin (*Clostridium perfringens*), botulinum toxin, produced by *Clostridium botulinum*, *Clostridium butyrricum*, *Clostridium barati*, and *Clostridium argentinensis*, and exotoxin A (ETA), produced by *Pseudomonas aeruginosa*, are discussed in the review of Dicks and Vermeulen [54]. Hormones secreted by endocrine cells control cell growth, metabolism, and response to environmental stress [55].

With aging, the immune system becomes impaired and fails to eliminate dead cells. This leads to the release of senescence-associated secretory phenotype (SASP). A typical example is an increase in the release of matrix metalloproteinase 9 (MMP-9) from aged VSMCs, which degrades the extracellular matrix, resulting in chronic inflammation [10,56,57]. The release of SASP factors from aged VSMCs induces the production of pro-inflammatory cytokines and stimulates foam cells to infiltrate arteries. Elevated levels of SASP and MMP-9 stimulate the lysis of collagen and the degradation of elastin, potentially causing plaque rupture and thrombosis [58]. For more information on immune changes with aging, the reader is referred to the review by Dicks [59].

This review does not attempt to cover all signaling molecules generated by gut microbiota. The interactions between signaling molecules and their receptors are discussed and evaluated as reporter systems in the early diagnosis of diseases. The reactions following these interactions are equally important to identify reporter molecules. The review focuses on a few prominent health conditions, i.e., metabolic syndrome (MetS), cancer, cardiovascular abnormalities, and mental disorders (MD). The biochemical and physiological conditions leading to these abnormalities will not be discussed. For further information on gastrointestinal disorders, cancer, cardiovascular diseases (CVDs), and MD, the reader is referred to the reviews by Dicks [1,10,13,14,16].

## 2. Metabolic Syndrome

**Figure 2:**
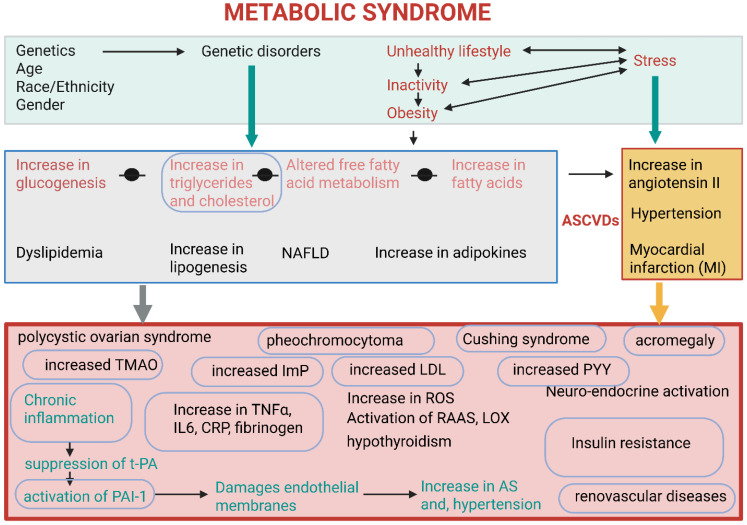
Multiple factors cause metabolic syndrome (MetS). Genetics, age, race/ethnicity, gender, and genetic disorders cannot be controlled. Some of these disorders may be of a metabolic nature (e.g., glucogenesis and the accumulation of triglycerides and fatty acids). An unhealthy lifestyle and factors associated with this may increase stress, vice versa, leading to more serious conditions such as hypertension and even heart failure. In the bottom rectangle are conditions associated with MetS, of which most are related to abnormalities of the inflammasome. Abnormalities encircled are key signals of MetS. TNFα = tumor necrosis factor alpha; IL6 = isoleucine 6; CRP = C-reactive protein; RAAS = renin–angiotensin–aldosterone; LOX = lysyl oxidase; ROS = reactive oxygen species; AS = atherosclerosis; ASCVDs = atherosclerotic cardiovascular diseases; t-PA = tissue plasminogen activators; PAI-1 = plasminogen activator 1; NAFLD = non-alcoholic fatty liver disease; LDL = low-density lipoprotein; PYY = peptide tyrosine-tyrosine; ImP = imidazole propionate; TMAO = trimethylamine N-oxide. This presentation was created in Biorender. LMT Dicks. (2025) https://app.biorender.com/illustrations/68d2909d543c60e859bc00c6 (accessed on 23 September 2025).

Metabolic syndrome (MetS) is the terminology used to describe several abnormalities, i.e., obesity, insulin resistance, hypertension, and abnormally high levels of TGAs and cholesterol (Figure 2). These conditions contribute to the development of atherosclerotic cardiovascular diseases (ASCVDs) and type II diabetes mellitus [60]. Individuals suffering from MetS have elevated levels of pro-inflammatory cytokines, e.g., tumor necrosis factor α (TNFα), and adipokines (proteinaceous hormones, cytokines, and growth factors) released by adipose tissue (Figure 2). Examples of the latter include leptin (controls food intake), adiponectin (exhibits anti-inflammatory properties), and resistin (regulates inflammation and insulin resistance) [61]. Adipokines secreted by adipose tissue act as messengers to control metabolic processes, inflammation, and immune responses throughout the body (Figure 2). They help regulate appetite, fat storage, glucose and fat metabolism, and blood pressure. Some adipokines, e.g., leptin and adiponectin, promote health, whilst pro-inflammatory cytokines such as TNF-α and IL-6 initiate inflammatory reactions [62]. High levels of TNF-α and interleukin-6 (IL-6) released from adipose tissue increase the risk of developing atherosclerosis (AS) [10,14], stimulate the liver to produce more C-reactive protein (CRP) [63], and increase blood pressure [64,65,66] (Figure 2). In summary, an abnormal increase in TNF-α and IL-6 signals chronic inflammation and insulin resistance, and may thus be strong indicators of MetS.

Abnormally high levels of pro-inflammatory cytokines may alter the endothelium from a relaxed to a prothrombotic state, suppress the production of tissue plasminogen activators (t-PA), and stimulate the production of plasminogen activator 1 (PAI-1) (Figure 2) [67]. t-PA converts the inactive precursor plasminogen into plasmin, which disintegrates blood clots [68]. PAI-1 suppresses the conversion of plasminogen into plasmin and prevents fibrinolysis, which leads to damage of endothelial membranes and the risk of developing AS and hypertension (Figure 2) [69,70]. PAI-1 is upregulated by angiotensin II (A-II), which is a key component of the renin–angiotensin–aldosterone (RAAS) [65]. t-PA and PAI-1 influence the fibrinolytic system and are strong signals of MetS. Elevated t-PA antigen levels are associated with insulin resistance, type 2 diabetes, obesity, and an increased risk of coronary heart disease (Figure 2).

Insulin resistance (hyperinsulinemia) is normally the first symptom of MetS [69]. Insulin resistance is associated with a high content of fecal monosaccharides, which correlates with an increase in *Lachnospiraceae* (*Dorea* and *Blautia*) and a decline in *Bacteroides* and *Alistipes* [70]. The authors have shown that feeding mice *Alistipes indistinctus* correlated with a decrease in blood glucose and fecal monosaccharides. Changes in cell numbers of *Dorea*, *Blautia*, *Bacteroides*, and *Alistipes* may thus be used to predict insulin resistance.

Other indications of MetS are elevated triglyceride levels, a decrease in high-density lipoprotein (HDL), an increase in low-density lipoprotein (LDL), renal dysfunction (renal parenchymal diseases, renovascular diseases), an increase in uric acid, abnormal liver functions, and an overactive thyroid (hypothyroidism) (Figure 2) [60]. Dietary triglycerides are enzymatically converted in the stomach and small intestine to release short-, medium- and long-chain fatty acids (SCFAs, MCFAs, and LCFAs, respectively), which are absorbed by the gut wall and used as an energy source [71]. The SCFAs acetate, propionate, and butyrate are primarily produced in the large intestinal tract by gut microbiota. Butyrate supplies 70% of the energy required by colonocytes [72], regulates glucose levels, and the production of most pro- and anti-inflammatory cytokines [1,10,14,73]. Several studies have shown that butyrate alleviates MetS by improving insulin sensitivity, enhancing lipid distribution, reducing chronic inflammation, and promoting gut barrier functions [1,14,73,74]. However, some studies have shown that butyrate worsens MetS by interacting with stearoyl CoA desaturase 1 (SCD-1) in the liver, where it converts saturated fatty acids to unsaturated fatty acids such as oleic and palmitoleic acids [1]. Butyrate levels may be a valuable method to evaluate the extent of MetS, and at the same time, manage MetS.

The accumulation of lipids in the liver, due to excessive plasma FFA, is characteristic of NAFLD [75]. This may develop into non-alcoholic steatohepatitis (NASH), cirrhosis [44], and type 2 diabetes [76]. Some individuals diagnosed with MetS develop polycystic ovarian syndrome, pheochromocytoma (excess catecholamine hormones produced from adrenal glands), Cushing syndrome (caused by excessive levels of the hormone cortisol), and acromegaly (excessive growth hormone production by benign tumors on the pituitary gland) (Figure 2). [77].

Central to all symptoms of MetS are the production of trimethylamine (TMA) from L-carnithine, which is oxidized in the liver into atherosclerogenic trimethylamine N-oxide (TMAO); imidazole propionate (ImP), which activates the mechanistic target of rapamycin complex 1 (mTORC1) pathway involved in insulin resistance; and aryl hydrocarbon receptor (AhR) agonists that activates AhR and regulates the immune system Figure 2 [78]. Dysbiosis and an increase in endotoxin-producing bacteria [79], and LPS levels [80] contribute to MetS. In mice, experimentally induced endotoxemia resulted in increased body weight, hyperglycemia, and hyperinsulinemia, similar to that observed in mice on a high-fat diet [80]. Findings from these studies have shown that signals produced by gut microbiota contribute to the development of MetS. Drastic changes in the levels of triglyceride, LDL, uric acid, C-reactive protein, catecholamine, cortisol, TMA, ImP, glucagon-like peptide 1 (GLP-1), peptide tyrosine-tyrosine (YY), IFN-γ, TLR2, and CD36 may serve as an early warning of MetS.

Irritable bowel syndrome (IBS), defined as one of the indicators of MetS, is linked to elevated triglycerides, LPS, Corticotropin-Releasing Factor (CRF), total cholesterol, and LDL cholesterol levels, abdominal obesity, stress, anxiety, depression, long-term antibiotic use, age, gender, diets, and increased waist circumference. Potential underlying pathophysiological commonalities include a compromised gut barrier and low-grade systemic inflammation [81,82]. Obese individuals are at a greater risk for chronic disease and often present with clinical parameters of metabolic syndrome (MetS), insulin resistance, and systemic markers of chronic low-grade inflammation [60]. The MAPK signaling pathway is closely involved in the development of insulin resistance. By dephosphorylating and deactivating multiple MAPKs, dual specificity phosphatase 9 restores the tyrosine phosphorylation level of insulin receptor substrate-1 (IRS1) and its capacity to mediate insulin signal transduction [83]. The signaling pathways involved in the pathogenesis of obesity, specifically in appetite regulation, adipose tissue metabolism and function, glucose hemostasis, and energy expenditure are summarized in the review by Wen et al. [84].

The causal relationship between gut microbiota modulation and metabolic syndrome is demonstrated through mechanisms such as the production of beneficial metabolites, and the reduction in inflammation. By modulating the gut microbiome through diets, prebiotics and probiotics, the gut barrier function improves, and inflammation is reduced. Beneficial microbes repair the gut lining and increase tight-junction proteins, preventing the transfer of microorganisms, toxins, LPS, etc., into the circulatory system. Metabolic compounds produced by gut microbiota act as signaling molecules that may influence host metabolic pathways, e.g., the regulation of insulin and glucose homeostasis.

## 3. Cancer

**Figure 3:**
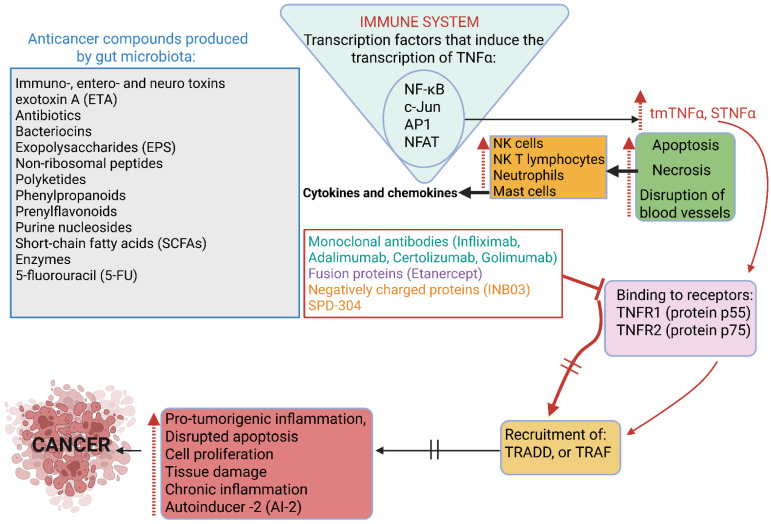
Anticancer compounds produced by gut microbiota are listed in the gray block. Cancer is synonymous with changes in our immune system (blue triangle), including the induction of transcription factors such as nuclear factor kappa B (NF-κB), c-Jun, activator protein 1 (AP-1), and nuclear factor associated with activated T lymphocytes (NFAT). The stimulation of these transcription factors leads to an increase in the transmembrane and soluble forms of tumor necrosis factor-alpha (tmTNFα and sTNFα, respectively). Cells are exposed to apoptosis, necrosis, and restricted blood flow (green block). These physiological changes stimulate the immune system (dark yellow block), leading to an increase in cytokines and chemokines. The binding of tmTNFα and sTNFα to TNFα protein receptors TNFR1 (protein p55) and TNFR2 (protein p75) (purple box), results in the recruitment of either TNFR1-associated death domain (TRADD) adaptor protein or TNFR-associated factor (TRAF) (light yellow box), followed by a series of reactions, e.g., pro-tumorigenic inflammation, disrupted apoptosis, cell proliferation, tissue damage, chronic inflammation, and an increase in autoinducer 2 (AI-2) (pink box), which manifests as cancer. Monoclonal antibodies, fusion proteins, negatively charged proteins, and the small TNFα inhibitor molecule SPD-304 prevent the binding of tmTNFα and sTNFα to TNFR1 and TNFR2, thus suppressing the activation of pro-tumorigenic reactions, apoptosis, cell proliferation, tissue damage, chronic inflammation, and autoinducer 2 (AI-2) production. This presentation was created in Biorender. LMT Dicks. (2025) https://app.biorender.com/illustrations/68d2afefffe0d4a45897c928 (accessed on 24 September 2025).

Cancer is one of the leading causes of death, according to 2022 statistics published by the American Cancer Society [85], with predictions that the number of cancer cases could increase to 21.6 million by 2030 [86]. Of all cancers, breast, lung, prostate, colon, rectum, bladder, kidney, renal, pelvis, pancreatic, thyroid, and liver cancer are the most common [87]. Several toxins, antibiotics, bacteriocins, non-ribosomal peptides, polyketides, phenylpropanoids, prenylflavonoids, purine nucleosides, short-chain fatty acids (SCFAs), and enzymes with anticancer properties, mostly produced by bacteria, have been described and discussed in several reviews [88]. (Figure 3). Some of these compounds are produced in response to signals generated by gut microbiota and tissue cells. The challenge is to identify these signals and develop methods to detect cancer at an early stage. This necessitates a better understanding of the interactions between anticancer drugs and their receptors.

The answer to finding a warning signal against cancer may lie in the reactions of our immune system. In the early 1900s W.B. Coley [89] noted that toxins (later referred to as Coley’s toxins) from heat-killed cells of *Streptococcus pyogenes* and *Serratia marcescens* raised the fever of patients and stimulated their immune system against tumors. It was later discovered that Coley’s toxins stimulated the production of tumor necrosis factor α (TNFα) [90,91,92,93]. An increase in TNFα induces apoptosis (programmed cell death) and necrosis (cell death due to injury), disrupts blood vessels, thus depriving tumor cells of blood supply (Figure 3). This activates natural killer (NK) cells, NK T lymphocytes, neutrophils, and mast cells to produce a series of cytokines and chemokines [94,95,96].

The expression of TNFα is induced by transcription factors such as NF-κB, c-Jun, activator protein 1 (AP1), and nuclear factor associated with activated T lymphocytes (NFAT) ((Figure 3) [97]. These transcription factors often function in a coordinated manner and are activated by stress and growth factors through the MAPK pathway. Both forms of TNF-α (tmTNF-α and sTNF-α) bind to TNF receptors TNFR1 (protein p55) and TNFR2 (protein p75), resulting in the recruitment of either TNFR1-associated death domain (TRADD) adaptor protein or TNFR-associated factor (TRAF) (Figure 3). This is followed by interactions with molecules from several complexes, resulting in the activation of inflammatory and apoptotic processes, cell proliferation, and host defense reactions (Figure 3). TNFR1 is expressed by all human cells, whereas TNFR2 is mainly expressed by immune cells [96,98]. Membrane-linked TNF-α or transmembrane TNF-α (tmTNF-α) and the processed form, soluble TNF-α (sTNF-α), are released from the membrane because of activated macrophages and lymphocytes, which induces the production of cytotoxic substances such as TNF-α.

Although tempting to predict cancerous growth by recording an increase in TNF-α levels, TNF-α is also produced by non-immune cells such as fibroblasts, endothelial cells, cardiac myocytes, and neurons, and is thus not always linked to immune responses [99,100]. Nevertheless, in some cancers (e.g., breast cancer), TNF-α promotes tumor formation [95] and may also cause autoimmune diseases such as multiple sclerosis, inflammatory bowel disease, rheumatoid arthritis, psoriatic arthritis, and systemic lupus erythematosus (SLE) [98]. Although inhibiting TNF-α by blocking its action with anti-TNF drugs such as monoclonal antibodies Infliximab, Adalimumab, Certolizumab, Golimumab, fusion proteins, e.g., Etanercept, and negatively charged proteins, such as the immune checkpoint inhibitor INB03 [95], and thus prevent binding to TNF receptors (TNFRs, (Figure 3), is a successful strategy for treating autoimmune and autoinflammatory diseases, the direct (and selective) blocking of TNFRs with specific agents is an alternative strategy. O’Connell et al. [100] described a small molecule inhibitor of TNF (SPD-304) that binds in a pocket within the trimer core, altering the conformation of the trimeric TNRF and rendering the TNF-TNFR interaction void. For further information, the reader is referred to McMillan et al. [101]. Applying the same approach to treat cancer may be challenging, as the surface of these cells is more negatively charged and their cell membranes are more fluid compared to healthy cells. It is, however, important to proceed with research on the interruption of TNFRs, as anti-TNF drugs cause severe side effects, e.g., an increase in infections, the risk of developing malignant tumors, and autoimmune conditions such as psoriasis, vasculitis, demyelinating disorders (disruption of nerve signals), and anti-TNF-induced lupus (ATIL) [102].

Several bacteria and microbial-produced bioactive compounds have been identified to treat cancer, reviewed by Baindara and Mandal [91] and Dicks [1,16]. More than a hundred clinical trials have been conducted to evaluate the anticancer properties of bacterial toxins (https://www.clinicaltrials.gov/, accessed on 1 June 2022), including the diphtheria toxin [103,104], enterotoxin CPE [105,106,107,108], botulinum toxin BoNT/A [109], and *Pseudomonas aeruginosa* toxin PE [110,111].

Antibiotics with anticancer properties are Actinomycin D (Dactinomycin, C_62_H_86_N_12_O_16_), Bleomycin A2 (C_55_H_84_N_17_O_21_S_3_), Bleomycin B2 (C_55_H_84_N_20_O_21_S_2_), Doxorubicin (adriamycin and doxil, C_27_H_29_NO_11_), Epirubicin (ellence, C_27_H_29_NO_11_), Cerubidine (Daunorubicin, DaunoXome, C_27_H_29_NO_10_), Novantrone (mitoxantrone, C_22_H_30_Cl_2_N_4_O_6_), Mitomycin C (C_15_H_18_N_4_O_5_), Spergualin (C_17_H_37_N_7_O_4_), Epothilone A (C_26_H_39_NO_6_S), Epothilone B (C_27_H_41_NO_6_S), and Epothilone D (C_27_H_41_NO_5_S), reviewed by Dicks and Vermeulen [54]. Bacteriocins described with anticancer properties are colicins (produced by *Escherichia coli*), plantaricin A (*Lactiplantibacillus plantarum* C11), microcin E492 (*Klebsiella pneumoniae*), and Pep27anal2 (*Streptococcus pneumoniae*), reviewed Dicks and Vermeulen [54]. Anticancer properties have also been reported for bovicin HC5 produced by *Streptococcus bovus* HC5, colicins A, E1, E3, and U isolated from *E. coli*, pyocins from *Pseudomonas aeruginosa*, nisin from *Lactococus lactis*, and pediocins from *Pediococcus* and other lactic acid bacteria, reviewed Dicks and Vermeulen [54]. Four enzymes with anticancer properties have been reported, i.e., arginine deiminase (ADI), produced by *Mycoplasma hominis* and *Mycoplasma arginine*, asparaginase (ASNase), produced by *E. coli* and *Erwinia chrysanthemi*, glutaminase and methionase, reviewed Dicks and Vermeulen [54].

The staggering amount of research invested in finding microbial-produced anticancer drugs is impressive. However, far less effort is spent on the receptors of these compounds on the surface of cancer cells. By focusing on a cure for cancer, the search for signaling molecules that may act as reporters of abnormal cell growth is neglected. Future research should be focused on the interaction of anticancer drugs with cancer cells and the composition of these receptors. Once the molecular structure of a receptor is unraveled and the mode of interaction with the anticancer drug is known, a cure may be found.

Elevated levels of autoinducer -2 (AI-2) were detected in tumors associated with colorectal cancer (CRC) [103]. This correlated with an increase in the expression of genes encoding TNFSF9 (tumor necrosis factor ligand superfamily member 9), as noted in tumor-associated macrophages [112]. AI-2 could thus be an important marker for CRC and warrants further research. Lung cancer antigen-1 (LC1) induces tumor growth factor beta (TGFβ), which is associated with tumor formation and colorectal cancer (CRC) [113].

Epithelial cellular adhesion molecule (Ep-CAM) keeps IECs in close contact, prevents mucosal infections, and maintains the selective function of the gut wall [114]. Changes in Ep-CAM levels affect the functioning of α (1,2) fucosyltransferase (FUT), which produces the A antigen by attaching fucose to the H antigen, and the B antigen by attaching a different sugar to the H antigen. The decrease in ABH antigens on the surface of cancer cells and an increase in sialyl-Lewis^(a)^ and sialyl-Lewis^(x)^ antigens promote metastasis by facilitating the adhesion of tumor cells to the endothelium and promoting the growth of tumor cells that resist apoptosis and spread to cells in other organs [115]. Sialyl-Lewis ^(a)^ and sialyl-Lewis^(x)^ antigens may thus act as signaling molecules to develop tumor-associated markers.

Most cancers originate from epithelial cells [116]. Squamous cell carcinomas (SCCs) are among the most prevalent human cancers and share similar mutation patterns, such as alterations in the *TP53*, *SOX2*, *TP63*, *CDNK2A* (*P16-INK4A*), *NOTCH1*, *KMT2D*, *PIK3CA* and *PTEN* genes [116]. The epithelial–mesenchymal transition (EMT) of cells determines their migration and invasive capabilities.

Further research is required to have a better understanding of the crosstalk between host AhR and bacterial QS molecules. An interesting advance in QS research in the last decade is the discovery that QSPs may promote tumor cell invasion and angiogenesis (at least in vitro), suggesting that these peptides may stimulate stem cell differentiation and the migration of cancer stem cells [117,118]. The influence microbiota has over colon cancer stem cells, “instructing” them to become treatable or non-treatable, was raised by Trosko and Lenz [119]. Some QS molecules stimulate tumor growth and are closely associated with the development of specific cancers, whilst others are linked to neurological disorders. QSPs that penetrate the blood–brain barrier (BBB) constitute an area that warrants more research, especially since the gut microbiome is increasingly recognized as a key player in neuropsychiatry.

## 4. Cardiovascular Functions

Cardiovascular disease (CVD) is mostly associated with an unhealthy diet, obesity, coronary heart disease, stroke, and diabetes, but may also be genetically inherited, which makes it a complex multifactorial disease. Associated with CVD are metabolic disorders, inflammation of, e.g., the gastrointestinal tract, liver, and CNS [10]. This defines cardiovascular abnormalities as a systemic disease rather than a heart disease. The multitude of factors leading to CVD are regulated by gut microbiota and include neurotransmitters (e.g., GABA; norepinephrine, NE; 5-HT and DA), SCFAs, glutamate (Glu), tryptophan (Trp), histamine (His), and secondary bile acids [10]. Efferent signals from the brain, channeled via the VN, reach enterochromaffin cells (ECs) and enteroendocrine cells (EECs) in the gut epithelium and mucosal layers. The GBA improves the integrity of the gut wall, reduces peripheral inflammation, and inhibits the release of pro-inflammatory cytokines [120]. Signals generated by the hypothalamus, in response to metabolites produced by gut microbiota, reach the pituitary and adrenal glands and communicate with EECs via the hypothalamic–pituitary–adrenal axis (HPA) [121]. The role that gut microbiota play in CVD is reviewed by Dicks [10].

Prolactin-releasing peptide (PrRP) activates neurons in the paraventricular nucleus (PVN) of the hypothalamus and increases the release of corticotropin-releasing hormone (CRH), suggesting that PrRP is involved in stress-related cardiovascular responses. PrRP regulates the sympathetic nervous system, blood pressure and heart rate [122], and, thus, has an impact on the pathophysiology of CVDs. PrRP binds to G protein-coupled receptor 10 (GPR10) and triggers various signaling cascades, including extracellular signal-regulated protein kinase (ERK), c-Jun N-terminal kinase (JNK), and phosphoinositide 3-kinase (PI3K)/Akt involved in cell growth, survival, and inflammation. [123,124].

**Figure 4:**
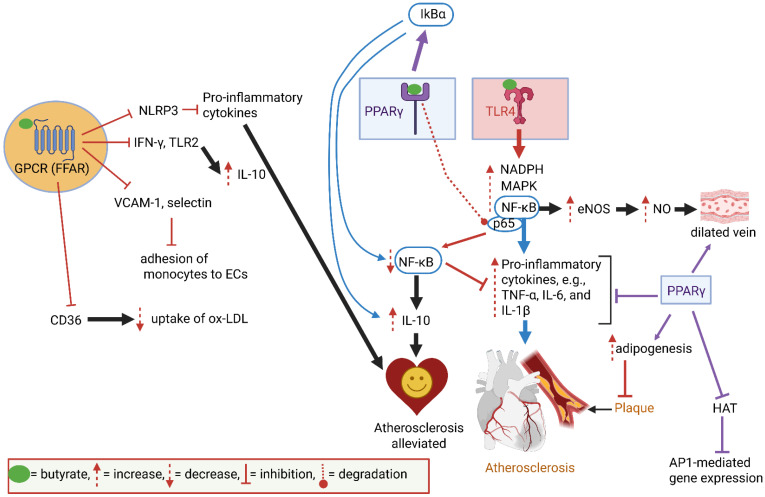
The binding of butyrate to Free fatty acid receptors (FFARs), also referred to as G protein-coupled receptors (GPCRs), suppresses the production of Nod-like receptor pyrin domain 3 (NLRP3), interferon gamma (IFN-γ), and Toll-like receptor 2 (TLR2), and downregulates the production of adhesion molecules such as vascular cell adhesion molecule 1 (VCAM-1) and E-selectin. The resultant suppression of pro-inflammatory cytokines prevents atherosclerosis (AS). The suppression of glycoprotein CD36 production prevents the uptake of oxidized low-density lipoprotein (ox-LDL) by macrophages. The interaction of butyrate with Toll-like receptor 4 (TLR4) leads to an increase in nicotinamide adenine dinucleotide phosphate (NADPH), mitogen-activated protein kinase (MAPK), and nuclear factor kappa B (NF-κB). The latter, when bound to protein p65, activates the expression of endothelial nitric oxide synthase (eNOS), resulting in an increase in nitric oxide (NO), which acts as a vasodilator. NF-κB stimulates the formation of pro-inflammatory cytokines, e.g., tumor necrosis factor alpha (TNF-α), isoleucine 6 (IL-6), and IL-1β, and initiates plaque formation. The production of pro-inflammatory cytokines is repressed when butyrate binds to peroxisome proliferator-activated receptor γ (PPARγ). The results in the degradation of p65, the inactivation of NF-κB, and the upregulation of anti-inflammatory cytokines such as IL-10. This suppresses atherosclerotic activities. PPARγ also induces adipogenesis and prevents the accumulation of lipids on atrial walls. ECs = endothelial cells; AP1 = activator protein-1; HAT = histone acetylase. The interactions between compounds are shown by using different colored arrows. The dotted line between PPARγ and p65 refers to degradation. This presentation was created in Biorender. LMT Dicks. (2025) https://app.biorender.com/illustrations/68d3d98964d8a647b368e9a7 (accessed on 24 September 2025).

The binding of butyrate to GPCRs suppresses the production of IFN-γ and TLR2, which increases the production of anti-inflammatory cytokines such as IL-10 (Figure 4). The suppression of NLRP3 by butyrate downregulates the production of pro-inflammatory cytokines (Figure 4) [10]. At the same time, the production of adhesion molecules such as vascular cell adhesion molecule 1 (VCAM-1) and E-selectin is suppressed, which prevents the adhesion of monocytes to ECs (Figure 4) [10]. Butyrate also suppresses the production of protein CD36, which is involved in the uptake of oxidized low-density lipoprotein (ox-LDL) by macrophages (Figure 4) [10]. CD36 acts as an ox-LDL scavenger, thus preventing the translocation of fatty acids to arterial walls. CD36 on the surface of foam cells accumulates advanced glycation end products (AGEs), which accelerate the formation of atherosclerotic plaques [10]. CD36 also stimulates blood clotting by aggregating platelets and binds to thrombospondin 1 (TSP-1), advanced oxidation protein products (AOPPs), and S100 family proteins (S100-A8, S100-A9, and S100-A12) to transfer ox-LDL into arteries [121,125]. The CD36-protein complex binds to Ca2+, growth hormone-releasing peptide (GHRP), cell-derived microparticles (MPs), and amyloids. If CD36 is compromised, as in damaged endothelia, plasma levels of Ca^2+^ and MPs (e.g., small vesicles that carry proteins and lipids) increase. The malfunctioning of GHRP disturbs the balance between growth hormone (GH) and insulin-like growth factor-1 (IGF-I). A modification of the GH/IGF-I axis may lead to the accumulation of lipids, an increase in body weight, and possibly also the development of AS [126]. Further research on the regulation of the GH/IGF-I axis may provide a better understanding of AS and CVDs. CD36 may be developed into a reporter system for the early detection of AS. Further research on the regulation of genes encoding CD36 and transcription factors such as PPAR may lead to the development of a ligand to detect CD36 levels. This is a challenging task, as CD36 is a diverse protein that binds to low-chain fatty acids (LCFAs), ox-LDL, thrombospondins, amyloid proteins, collagen, and AGE.

Activation of TLR4 by butyrate increases NF-κB, NADPH, and MAPK (Figure 4) [10]. NF-κB activates the expression of eNOS, leading to an increase in NO levels, and stimulates the formation of pro-inflammatory cytokines (e.g., TNF-α, IL-6, and IL-1β) [10]. (Figure 4). These cytokines initiate plaque formation in arteries. The binding of butyrate to PPARγ induces the activity of IkBα, an inhibitor of the NF-κB pathway. (Figure 4). The downregulation of NF-κB increases the production of anti-inflammatory cytokines, e.g., IL-10 [10]. Thus, by binding to PPARγ, butyrate indirectly suppresses plaque formation (Figure 4). PPARγ also induces adipogenesis, thus preventing the accumulation of lipids on atrial walls [10].

Most cardiovascular abnormalities develop over years and are often only detected with sudden heart failure, a heart attack, or stroke. Several abnormalities on a molecular level may, however, serve as warning signals. Butyrate regulates AS [10]. It thus makes sense to focus on receptors, such as PPAR, TLR and AhR, butyrate-influenced signal-generating pathways, such as MAPK and NLRP3 inflammasome, and ox-LDL transporters. Furthermore, by studying changes in immune reactions, we may identify unique signaling molecules to detect AS much earlier. Biomarkers that may be considered for the early detection of AS are: The epithelial cellular adhesion molecule (Ep-CAM), trefoil factor 3 (TF3), leptin, plasminogen activator inhibitor 1 (PAI-1), alpha-1 acid glycoprotein 1 (AGP1), contactin 1 (CNTN1), butyrate, TMAO, NLRP3, and elastin. Butyrate regulates AS. It is thus sensible to focus on receptors, such as PPAR, TLR, AhR, and butyrate-influenced signal-generating pathways, such as MAPK, and ox-LDL transporters to find a cure for AS. Our knowledge of the interactions between butyrate and receptors on IECs and ECs, specifically GPCRs (free fatty acid receptors, FFARs), provides a solid basis for the intervention of AS and may lead to the design of novel GPCR-targeted drugs.

In summary, gut microbiota modulate cardiovascular health through mechanisms such as reducing inflammation, improving glucose and lipid metabolism, and altering the production of harmful metabolites such as TMAO and phenylacetylglutamine (PAGln). The latter increases platelet aggregation and increases the risk of thrombosis [127]. For further information on the association between gut microbiota signaling molecules and CVDs, the reader is referred to the reviews by Dicks [10,14,58,59].

## 5. Brain Functions

**Figure 5:**
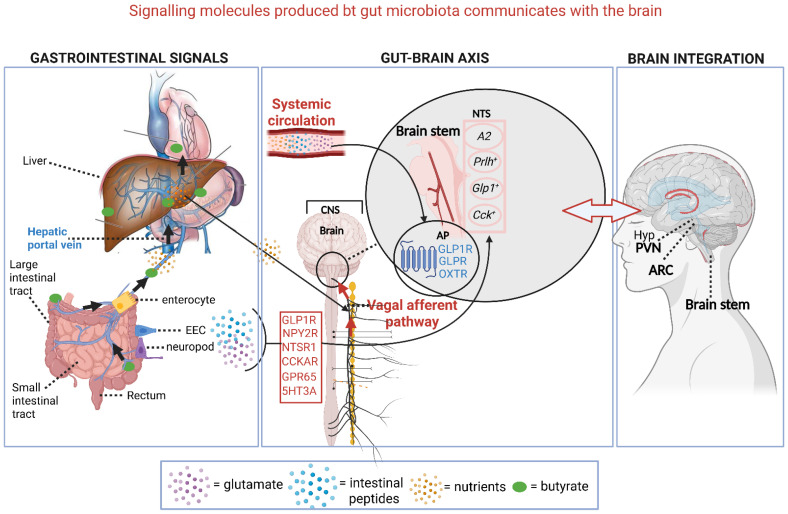
Gastrointestinal signals generated by gut microbiota interact with enterocytes, enteroendocrine cells (EECs), and neuropods in the intestinal epithelium (left section). Enterocytes absorb nutrients, whilst EECs detect luminal signals and release hormones to regulate metabolism and gut functions. Neuropods rapidly relay signals to the nervous system and reach the elongated nucleus tractus solitarius (NTS). Neurons in the forebrain and hypothalamus (hyp) interact with the NTS and control sensorial, endocrine, social, emotional, stress-, and learning-related functions. NTS neurons also connect with the arcuate nucleus of the hypothalamus (ARC) (right section), and transfer signals that respond to glucoprivation. Signals from the NTS and the brainstem reach the hypothalamic paraventricular nucleus (PVN) (right image), which regulates cardiovascular functions such as hypoxia, blood pressure, the release of hormones (e.g., oxytocin and vasopressin), and satiety. CNS = central nervous system; GLP1R = Glucagon-like peptide-1 receptor; NPY2R = neuropeptide PYY receptor; NTSR1 = neurotensin receptor 1; CCKAR = cholecystokinin A receptor, (CCKAR); GPR65 = G protein-coupled receptor 65; 5HT3A = 5-hydroxytryptamine (serotonin) receptor 3A; OXTR = neurotransmitter oxytocin receptor; *A2*, *Prlh^+^*, *Glp1^+^*, and *Cck^+^* 1 = NTS neurons. This presentation was created in Biorender. LMT Dicks. (2025) https://app.biorender.com/illustrations/68838fb39f7be53543cb9e4d (accessed on 24 September 2025).

Gastrointestinal signals generated by gut microbiota (e.g., butyrate, glutamate, peptides, metabolites) interact with enterocytes, EECs, and neuropods in the intestinal epithelium (left section, Figure 5). Enterocytes absorb nutrients, whilst EECs detect luminal signals and release hormones to regulate metabolism and gut functions. Neuropods rapidly relay signals to the nervous system. Cell bodies of these neurons, found in the nodose ganglia (NG), play a key role in metabolic regulation. Glucagon-like peptide-1 receptor (GLP1R) neurons stimulate muscle layers of the stomach, distant from GLP-1-secreting gut cells [113]. Other receptors are the cholecystokinin A receptor (CCKAR), neuropeptide PYY receptor (NPY2R), neurotensin receptor 1 (NTSR1), G protein-coupled receptor 65 (GPR65), and 5-hydroxytryptamine (serotonin) receptor 3A (5HT3A) (middle section, Figure 5) [128].

Signaling molecules cross the gut–blood barrier or are transferred to the circulatory system via the hepatic portal vein (left section, Figure 5). All signals reach the CNS via afferent fibers of the Vagus nerve, which enter the brain through the brainstem, where they synapse onto elongated nucleus tractus solitarius (NTS) neurons *A2*, *Prlh^+^*, *Glp1^+^*, and *Cck^+^* (middle section, Figure 5) [129]. Molecules in the circulatory system interact with G protein-coupled receptors (GPCRs), such as GLPR1, GLPR, and OXTR (middle section, Figure 5), on the surface of arterial endothelial cells, nuclei, and glial cells of the brainstem [16].

A complex arrangement of nerves branching from the Vagus nerve controls metabolic and digestive processes. Neurons in the forebrain and hypothalamus (hyp, right section Figure 5) interact with the NTS and control sensorial, endocrine, social, emotional, stress-, and learn-related functions [114]. NTS neurons also connect with the arcuate nucleus of the hypothalamus (ARC) (right section, Figure 5), and transfer signals that respond to glucoprivation [130,131]. Signals from the NTS and the brainstem reach the hypothalamic paraventricular nucleus (PVN) (right image, Figure 5), which regulates cardiovascular functions such as hypoxia, blood pressure, the release of hormones (e.g., oxytocin and vasopressin), and satiety [129,132].

Gut microbiota has an immense impact on the GBA and overall mental health, as reviewed by Dicks et al. [16]. GABA and metabolites secreted by gut microbiota have an important impact on anti-inflammatory responses and relieve psychiatric symptoms stemming from inflammation [16,133]. Treatment of schizophrenic and bipolar patients with probiotics alleviated symptoms associated with IBD, autistic children benefited from probiotic treatment, and OCD-like behavior could be controlled [16].

Our understanding of gut microbiota controlling cognitive behavior, mood, and neuropsychiatric disorders is limited and requires in-depth study to decipher the complex network between cells and neurons. Extensive and carefully controlled clinical trials need to be conducted to evaluate the effectiveness in treating mental disorders.

Interactions between drugs and gut microbiota need to be studied in greater depth and will have to include multi-omics of gut and oral microbiota, and more research on quorum sensing. This includes interactions between gut microbiota and gut epithelial cells. The synthesis, regulation, and production of serotonin and other neurotransmitters, e.g., glutamine (Glu), GABA, DA, norepinephrine, and histamine by gut microbiota requires more research [15]. This includes studies on signals transported via the Vagus nerve, autonomic sympathetic and parasympathetic nervous systems, and the hypothalamic–pituitary–adrenal axis (HPA) [13]. In-depth studies must be conducted on the effect of SCFAs, tryptophan, and secondary bile acids on the CNS [16]. We need to have a better understanding of the influence gut microbiota have on the forming of new neurons. The focus should be on gut microbiota with the ability to produce and metabolize hormones [15]. These studies are important, as minor activation of neurons results in drastic changes in the production of neurotransmitters, which affects digestion, intestinal permeability, gastric motility, and immune regulation [134]. GABA, in addition to other metabolites, plays an important role in anti-inflammatory responses and helps alleviate psychiatric symptoms stemming from inflammation [16]. Treatment of schizophrenic and bipolar patients with probiotics alleviated symptoms associated with IBD, and autistic children benefited from probiotic treatment. Obsessive–compulsive disorder (OCD)-like behavior could also be controlled by treatment with LAB.

Our understanding of the influence gut microorganisms has on cognitive behavior, mood, and neuropsychiatric disorders remains limited. By studying the gut microbiome, QS, neurotransmitters, and the GBA, on a molecular level, the greater the chance of developing novel therapeutics, probiotics, and psychobiotics. This calls for in-depth deciphering of the complex, ever-changing network between cells and neurons. Research on the quenching of QS signals needs to be prioritized. We need to understand how quorum quenching (QQ) therapy will affect beneficial gut microbiota. Based on these findings, biomarkers could be developed to identify differences in the gut microbiome of individuals suffering from psychological disorders. This will also allow us to understand the effect psychiatric medication may have on the composition of the gut microbiome. We need to determine at which level gut microbiota metabolize drugs. Studies should include multi-omics of gut and oral microbiota to have a better understanding of the mutual interplay between phyla. Will it be possible to develop probiotics to treat dysbiosis and neuropsychiatric abnormalities?

From the data published, it is clear that gut microbiota can modulate brain functions through the brain–gut-microbiome axis. These interactions influence neuronal, immune, and endocrine pathways. Modulation of the gut microbiome (e.g., through diet or probiotics) can improve cognitive functions, mood, stress, and anxiety-like behaviors. The role gut bacteria play in neuropsychiatric disorders, supported by clinical studies, is reviewed by Dicks et al. [16]. The authors highlighted the importance of deciphering the complex, ever-changing network between microbial cells and neurons. Most of the studies have been performed on animals. Although preclinical and clinical investigations have shown that treatment with specific strains of *Lactococcus*, *Lactobacillus*, *Streptococcus*, *Morganella*, *Klebsiella*, *Hafnia*, *Bacteroides*, *Bifidobacterium*, *Propionibacterium*, *Eubacterium*, *Roseburia* and *Prevotella* may influence mental behavior, extensive and carefully controlled clinical trials are scarce [15]. More longitudinal studies are needed to determine if medications taken for psychiatric and neurological disorders alter the composition of the gut microbiome. For further information on gut bacteria, neuropsychiatric disorders and neurotransmitters, the reader to the reviews by Dicks et al. [16] and Dicks [15].

## 6. Conclusions

Gut microbiota regulate several physiological processes via signaling molecules such as neurotransmitters, gamma-aminobutyric acid (GABA), short-chain fatty acids (SCFAs), glucagon-like peptide 1 (GLP-1), peptide tyrosine-tyrosine (PYY), and cholecystokinin (CCK). Key signals produced by gut microbiota that are linked to metabolic syndrome (MetS), cancer, cardiovascular diseases (CVDs), and brain functions are listed in Table 1. These signaling molecules interact with pattern recognition receptors (PRRs), such as Toll-like receptors (TLRs), proliferator-activated receptors (PPARs), damage-associated molecular patterns (DAMPs), free fatty acid receptors (FFARs), proliferator-activated receptors (PPARs), and aryl hydrocarbon receptors (AhRs) that are embedded into intestinal epithelial cells (IECs), enteroendocrine cells (EECs), endothelial cells (ECs), and macrophages. The ligand–receptor complexes stimulate a cascade of downstream reactions that regulate immune and inflammatory responses, intestinal mobility, the release of hormones, and neurological functions. Signals reach the central nervous system (CNS), either directly via the Vagus nerve or indirectly via the enteric nervous system (ENS). The exchange of signals via the gut–brain axis (GBA) keeps the gut microbiota in a balanced state and prevents dysbiosis that may result in gastrointestinal disorders such as Crohn’s disease, ulcerative colitis (UC), and celiac disease. A select group of signals maintains liver functions (e.g., glucose and lipid metabolism) and regulates complex enteroendocrine interactions. Of all signaling molecules discussed here, the SCFA butyrate plays a pivotal role in metabolic syndrome (MetS), and may hold the answer to the prevention, or even treatment, of cancer. Other compounds from gut microbiota that exert anticancer properties are toxins, antibiotics, bacteriocins, non-ribosomal peptides, polyketides, phenylpropanoids, phenylflavonoids, and purine nucleosides. Butyrate holds a central position in cardiovascular diseases (CVDs). In this case, the interaction of butyrate with free fatty acid receptors (FFARs), proliferator-activated receptors (PPARs), TLRs, and aryl hydrocarbon receptors (AhRs) influences signal-generating pathways such as mitogen-activated protein kinase (MAPK) and Nod-like receptor pyrin domain 3 (NLRP3). Protein CD36 regulates the uptake of oxidized low-density lipoprotein (ox-LDL) by macrophages. By modulating the chemical structure of butyrate receptors, we may increase the sensitivity of cells to cardiovascular drugs. Possible biomarkers for the early detection of CVDs include SCFAs, epithelial cellular adhesion molecule (Ep-CAM), trefoil factor 3 (TFF3) peptide, leptin, plasminogen activator inhibitor 1 (PAI-1), alpha-1 acid glycoprotein 1 (AGP1), contactin 1 (CNTN1), trimethylamine N-oxide (TMAO), NLRP3, elastin, prostacyclin (PGI2), angiotensin II, bradykinin (a vasodilator), apolipoprotein D-F4, protein p53, cell cycle arrest protein P21, endothelial nitric oxide synthase (eNOS), and matrix metalloproteinase 9 (MMP-9). Butyrate produced by gut microbiota influences our mental health. GABA plays an important role in anti-inflammatory responses and helps alleviate psychiatric symptoms stemming from inflammation. The deciphering of this complex, ever-changing network between cells and neurons requires in-depth research that involves metagenomics, metabolomics, and proteomics.

## Figures and Tables

**Table 1:** Key signals produced by gut microbiota that are linked to metabolic syndrome, cancer, cardiovascular diseases, and brain functions.

Abnormality	Key Signals and Gut Microbiota ^a^	Physiological Effects	References
**Metabolic Syndrome**	**Short-chain fatty acids (SCFAs).** *Faecalibacterium*, *Eubacterium*, *Bacteroidetes*, *Phascolarctobacterium*, *Veillonella*, *Lachnospiraceae*, *Ruminococcaceae*, *Bifidobacterium*, *Lactobacillus*, *Blautia*, *Coprococcus*, *Roseburia*, *Clostridium*	Improve insulin sensitivity, regulate lipid metabolism, inhibit histone deacetylases (HDACs), reduce cholesterol synthesis, serve as an energy source for colonocytes, anti-inflammatory, improve gut peristalsis, promote neurogenesis and synaptic plasticity, have pro- and anticancer effects (depending on concentration)	[1,14,25,60,81,82]
	**Bile acids (BAs)**, e.g., deoxycholic acid (DCA) and taurine-conjugated BAs. *Clostridium*, *Bacteroides*, and *Eubacterium*	Metabolized by gut microbiota into secondary BAs that impact lipid and glucose metabolism. DCA is associated with inflammation and colorectal carcinogenesis by activating specific signaling pathways, such as mitogen-activated protein kinase (MAPK). Dysregulation of BAs can contribute to neurological dysfunction	[1,14,45,48]
	**Tryptophan (Trp) metabolites**, e.g., indole-3-propionic acid (IPA), and indole. *Lactobacillus*, *Bifidobacterium*, *Clostridium*, *Ruminococcus*	Trp is a precursor to serotonin (5-HT). Dysregulation of Trp metabolism is linked to anxiety and depression. IPA and indoles activate aryl hydrocarbon receptors (AhRs), which regulate metabolism and reduce inflammation. Lower levels of these metabolites are linked to insulin resistance. Indoles suppress cancer progression by activating AhR	[1,21,22,30,50]
	**Trimethylamine N-oxide (TMAO).** Generated from trimethylamine (TMA) by the liver. TMA is produced by gut bacteria metabolizing choline and carnitine. *Enterobacteriaceae*, *Lactobacillus*, *Clostridium*.	High levels of TMAO are associated with insulin resistance and type 2 diabetes, atherosclerosis (AS), endothelial dysfunction, heart failure, certain cancers, and neuroinflammation	[78,127]
	**Lipopolysaccharides (LPSs).** Mainly Gram-negative bacteria, e.g., *Escherichia coli*, *Bacteroides*, *Akkermansia*, Proteobacteria	Causes increased intestinal permeability (leaky gut), triggers chronic inflammation, and insulin resistance. Promote inflammation-related carcinogenesis by activating inflammatory pathways, such as nuclear factor kappa B (NF-κB), and induce DNA damage. Chronic systemic inflammation impairs endothelial wall function and promotes AS. Causes neuroinflammation when the blood–brain barrier is crossed	[27,28,80,81,82]
	**Gamma-amino butyric acid (GABA).** *Bifidobacterium*, *Lactobacillus*, *Bacteroides*, *Akkermansia*	Primary inhibitory neurotransmitter in the central nervous system (CNS), affecting mood and anxiety. Modulates intestinal motility	[10,15,16]
	**Serotonin (5-HT).** *Enterococcus*, *Streptococcus*, *Escherichia*	Enterochromaffin cells produce most of the body’s 5-HT, but gut bacteria can produce it directly or stimulate its production. Regulates gut motility and is a key signaling molecule in the gut–brain axis (GBA)	[19,27]
	**Dopamine.** *Enterococcus*, *Eubacterium*, *Blautia*, *Bacillus*, *Staphylococcus*, *Escherichia*, *Serratia*	A neurotransmitter, crucial for motor control, motivation, pleasure, reward, and cognitive functions such as learning, memory, and attention	[49]
	**Gut hormones**, e.g., glucagon-like peptide-1 (GLP-1), peptide tyrosine-tyrosine (peptide YY), and cholecystokinin (CCK). Produced by intestinal epithelial cells (IECs)	Suppresses appetite and decreases food intake by signaling satiety from the gut to the brain	[11,35]
	**Ankyrin.** Not produced by gut microbiota	Initiates inflammatory responses by releasing neuropeptides that stimulate immune cells. Plays a role in tissue repair and intestinal motility	[19,22]
	**Toxins**, e.g., fragilysin (BFT), uremic toxin, diphtheria toxin, enterotoxin, botulinum toxin, exotoxin A. *Bacteroides fragilis*, *Clostridium diphtheriae*, *Clostridium perfringens*, *Clostridium botulinum*, *Clostridium butyrricum*, *Clostridium barati*, *Clostridium argentinensis*, *Pseudomonas aeruginosa*	Damage tight junction proteins, increase gut permeability, and cause inflammation	[13,53,54]
**Cancer:**	**Secondary BAs,** e.g., deoxycholic acid (DCA) and lithocholic acid (LCA). *Clostridium*, *Bacteroides*, *Eubacterium*, *Lactobacillus*, *Bifidobacterium*	Elevated levels linked to colon cancer	[1,14,45,48]
	**Bacterial toxins.** See microbiota listed under “Toxins”	Interact with host cells, alter physiological functions, and promote tumor growth	[91,92]
**Cardiovascular Diseases (CVDs):**	**Trimethylamine N-oxide (TMAO).** *Enterobacteriaceae*, *Lactobacillus*, *Clostridium*	Produced from choline and carnitine, linked to vascular inflammation and endothelial damage	[78,127]
	**BAs.** *Clostridium*, *Bacteroides*, and *Eubacterium*	Affect cardiac muscle function, and influence lipid metabolism and plaque formation	[1,14,45,48]
	**Inflammatory mediators.** *Escherichia coli*, *Bacteroides*, *Akkermansia*, Proteobacteria	Lipopolysaccharides (LPSs) trigger inflammation	[27,28,80,81,82]
	**Olfactory receptor OR51E1** (Olfr588). Produced by IECs	Influences blood pressure, vascular reactivity, and arterial stiffness	[25]
	**Transient receptor potential ankyrin A1 (Trpa1).** Not produced by gut microbiota	Increases intestinal motility and the transfer of Ca^2+^	[20,21,22]
	**Oleoylethanolamide (OEA), palmitoylethanolamide (PEA), lysophosphatidylcholine (LPC).** Not produced by gut microbiota	Regulate the release of glucagon-like peptide-1 (GLP-1) and peptide tyrosine-tyrosine (PYY), which control food intake. The activation of the brainstem nucleus tractus solitarius (NTS) by GLP-1 influences mood, cognition, and gastrointestinal motility	[1,24,30]
	**Isovaleric acid.** *Bacteroides*, *Clostridium* **Senescence-associated secretory phenotype (SASP)**, e.g., matrix metalloproteinase 9 (MMP-9). Not produced by gut microbiota	An intermediate in the synthesis of cholesterol and fatty acids. Interacts with olfactory receptor OR51E1 (equivalent to olfactory receptor 558, Olfr588, in mice). Regulates blood pressure, vascular reactivity, and arterial stiffness	[23,25]
	**Senescence-associated secretory phenotype (SASP)**, e.g., matrix metalloproteinase 9 (MMP-9). Not produced by gut microbiota	Degrades the extracellular matrix, resulting in chronic inflammation	[10,56,57,58,59]
**Brain Functions:**	**5-HT.** *Enterococcus*, *Streptococcus*, *Escherichia*	The majority of 5-HT is produced in the intestine. Gut microbiota influences the levels produced. Impact on mood	[30,31,32,33,49,50,128]
	**GABA.** *Bifidobacterium*, *Lactobacillus*, *Bacteroides*, *Akkermansia*	Produced by some microorganisms. A neurotransmitter that affects emotions	[10,15,16,133]
	**Inflammatory molecules.** *Escherichia coli*, *Bacteroides*, *Akkermansia*, Proteobacteria	LPS triggers cytokine production (e.g., TNF-α, IL-6), which affects brain function. Associated with anxiety, depression, and memory	[1,14,28,33]
	**Polysaccharide A (PSA).** *Bacillus fragilis*	May protect against CNS inflammation	[28,52]

^a^ Main gut microbiota are listed as producers of signals, or involved in the activation of signaling molecules.

## Data Availability

No new data were created or analyzed in this study. Data sharing is not applicable to this article.
